# PREDICTORS OF HEALTH-RELATED QUALITY OF LIFE AND RECOVERY AMONG OLDER ADULTS WITH SERIOUS MENTAL ILLNESS

**DOI:** 10.1093/geroni/igz038.3215

**Published:** 2019-11-08

**Authors:** Anjana Muralidharan, Clayton H Brown, Richard W Goldberg

**Affiliations:** 1 Veterans Affairs Capitol Healthcare Network, Mental Illness Research Education and Clinical Center, Baltimore, Maryland, United States; 2 University of Maryland School of Medicine, Baltimore, Maryland, United States; 3 Veterans Affairs Capitol Healthcare Network, Baltimore, Maryland, United States

## Abstract

Older adults with serious mental illness (i.e., schizophrenia spectrum disorders and affective psychoses) exhibit marked impairments across medical, cognitive, and psychiatric domains. The present study examined predictors of health-related quality-of-life and mental health recovery in this population. Participants (N=211) were ages 50 and older with a chart diagnosis of serious mental illness and a co-occurring medical condition, engaged in outpatient mental health services at a study site. Participants completed a battery of assessments including subtests from the Repeatable Battery for the Assessment of Neuropsychological Status (RBANS), the 24-Item Behavior and Symptom Identification Scale (BASIS-24), the 12-Item Short-Form Health Survey (SF-12), and the Maryland Assessment of Recovery Scale (MARS). Multiple linear regression analyses, with age, race, gender, and BMI as covariates, examined number of current medical conditions, RBANS, and BASIS as predictors of quality-of-life and recovery. Significant predictors of physical health-related quality-of-life (R-squared=.298, F(9,182)=8.57, p<.0001) were number of medical conditions (β=-1.70, p<.0001), BASIS-Depression/Functioning (β=-4.84, p<.0001), and BASIS-Psychosis (β=2.39, p<.0008). Significant predictors of mental health-related quality-of-life (R-squared=.575, F(9,182)=27.37, p<.0001) were RBANS (β=0.03, p=.05), BASIS-Depression/Functioning (β=-6.49, p<.0001), BASIS-Relationships (β=-3.17, p<.0001), and BASIS-Psychosis (β=-1.30, p=.03). Significant predictors of MARS (R-squared=.434, F(9,183)=15.56, p<.0001) were BASIS-Depression/Functioning (β=-4.68, p=.002) and BASIS-Relationships (β=-9.44, p<.0001). To promote holistic recovery among older adults with serious mental illness, integrated interventions are required. For example, to improve physical health-related quality-of-life, one should target depression and psychotic symptoms as well as medical illness burden. To improve mental health-related quality-of-life, depression symptoms and interpersonal functioning may be key targets, as well as neurocognitive function.

